# NMR cryoporometry characterisation studies of the relation between drug release profile and pore structural evolution of polymeric nanoparticles

**DOI:** 10.1016/j.ijpharm.2014.04.018

**Published:** 2014-07-20

**Authors:** Navin Gopinathan, Bin Yang, John P. Lowe, Karen J. Edler, Sean P. Rigby

**Affiliations:** a Department of Chemical and Environmental Engineering, University of Nottingham, University Park, Nottingham NG7 2RD, UK; b Department of Chemistry, University of Bath, Claverton Down, Bath BA2 7AY, UK

**Keywords:** Drug, Controlled release, PLGA, Diffusion, Cryoporometry, NMR

## Abstract

PLGA/PLA polymeric nanoparticles could potentially enhance the effectiveness of convective delivery of drugs, such as carboplatin, to the brain, by enabling a more sustained dosage over a longer time than otherwise possible. However, the link between the controlled release nanoparticle synthesis route, and the subsequent drug release profile obtained, is not well-understood, which hinders design of synthesis routes and availability of suitable nanoparticles. In particular, despite pore structure evolution often forming a key aspect of past theories of the physical mechanism by which a particular drug release profile is obtained, these theories have not been independently tested and validated against pore structural information. Such validation is required for intelligent synthesis design, and NMR cryoporometry can supply the requisite information. Unlike conventional pore characterisation techniques, NMR cryoporometry permits the investigation of porous particles in the wet state. NMR cryoporometry has thus enabled the detailed study of the evolving, nanoscale structure of nanoparticles during drug release, and thus related pore structure to drug release profile in a way not done previously for nanoparticles. Nanoparticles with different types of carboplatin drug release profiles were compared, including burst release, and various forms of delayed release. ESEM and TEM images of these nanoparticles also provided supporting data showing the rapid initial evolution of some nanoparticles. Different stages, within a complex, varying drug release profile, were found to be associated with particular types of changes in the nanostructure which could be distinguished by NMR. For a core-coat nanoparticle formulation, the development of smaller nanopores, following an extended induction period with no structural change, was associated with the onset of substantial drug release. This information could be used to independently validate the rationale for a particular synthesis method. Hence, the specific reasons for the effectiveness of the synthesis route, for obtaining core-coat nanoparticles with delayed release, have been elucidated.

## Graphical abstract

Nanoparticles for convective delivery.

**Figure:**
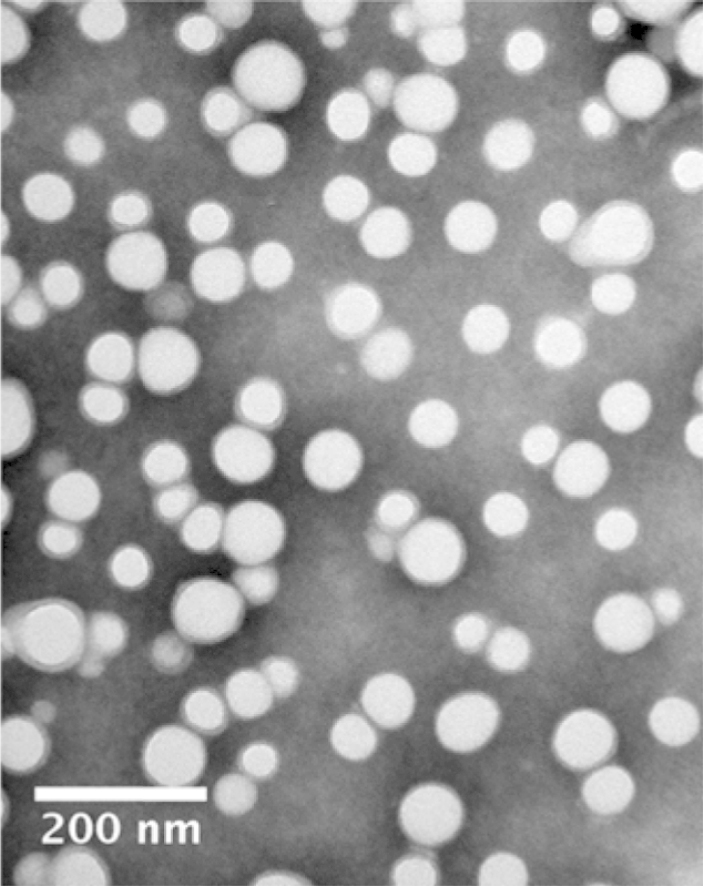

## 1 Introduction

It is well known that drug therapy can be improved by the use of controlled release systems. Time controlled delivery is essential for many different drugs, especially chemotherapeutics, as a spike in the concentration of the drug can cause major side effects. Further, in some instances, controlled release systems also provide a means to increase patient compliance and convenience. They help in maintaining the required drug concentration levels within the body without undergoing rapid clearance. Recent studies (Averineni et al., 2012; Avgoustakis et al., 2002; Gryparis et al., 2007; Hammady et al., 2009; Martinelli et al., 2011; Morgen et al., 2012; Rescignano et al., 2012; Sant et al., 2005; Sawyer et al., 2011; Soppimath et al., 2001; Valot et al., 2010) have revolved around the use of biocompatible and biodegradable polymeric nanoparticles that serve as carriers transporting drugs to the site of action. These nanoparticles are usually synthesised by dispersing a polymer in an organic solvent followed by transfer into a non-solvent, or by direct polymerisation of monomers. Different synthesis routes are available, allowing the synthesis of different kinds of nanoparticles having unique properties, *i.e.*, they may have a low or high drug loading, narrow or broad size distribution, *etc*. These properties result in specific drug release profiles following various mechanisms (Lu et al., 2011; Soppimath et al., 2001; Vilar et al., 2012). Mechanisms could include desorption of the surface bound drug, diffusion through the polymer pore network, release controlled by the fracture/degradation of the polymer or a combination of diffusion and degradation. The drug release profiles depend on whether or not the drug is uniformly distributed in the polymer matrix. Generally, drug release by diffusion takes place when the polymer matrix degrades at a far slower rate. On the other hand, rapid initial or burst release is often attributed to the drug fraction which is adsorbed or weakly bound to the large surface area of the polymer nanoparticles. Thus, drug release rates of polymer nanoparticles depend on the physical and chemical structure of the polymer and its interaction with drug molecules (Lu et al., 2011; Soppimath et al., 2001; Vilar et al., 2012).

The release patterns obtained depend on the nanoparticle formulation. Time-controlled formulations allow the drug to reach therapeutic concentrations in the tissue before gradually tailing off. When chronic treatments are required, similar doses are administered at regular time intervals to maintain drug concentrations within the therapeutic window. Unlike time-controlled formulations, modified release formulations deliver the drug at a defined speed or site of interest. Such formulations can be classified depending on the drug release patterns. They include extended release formulations, sustained release formulations, pulsatile release formulations and delayed release formulations (Vilar et al., 2012). Table 1 features the details regarding different formulations.

From Table 1, it can be seen that modified release formulations provide alternatives to routine drug administration and improve therapeutic action. They are highly beneficial for drugs that show poor bioavailability or those with a narrow therapeutic window. They are also useful for drugs that are unstable in certain environments or where degree of compliance is low. However, major disadvantages of these formulations are their high production costs, lack of reproducibility, unpredictable *in vitro*-*in vivo* correlations and problems associated with improper handling.

Nanoparticle properties are determined with the aid of different characterisation techniques and by studying their drug release profiles. In the literature, nanoparticle characterisation is primarily concerned with the determination of particle size, size distribution, particle morphology and zeta (ζ) potential. Particle size and size distribution are determined using dynamic light scattering (DLS) or laser diffraction (LD). The size distribution is usually indicated in the form of a polydispersity index. A low value (0.1–0.25) indicates a narrow size distribution while larger values (>0.5) refer to a broad size distribution. Particle morphology is usually determined using different microscopy techniques, namely scanning electron microscopy (SEM), transmission electron microscopy (TEM) and atomic force microscopy (AFM). However, the disadvantage of microscopy techniques are that the information obtained is, potentially, only characteristic of the particular section of the sample observed. They need not be representative of the entire batch. Another property of interest is ζ potential which is obtained using Laser Doppler electrophoresis. It measures the electrophoretic mobility of the nanoparticles suspended in a medium and provides a measure of the stability of particles within a medium. A ζ potential value >60 mV indicates excellent stability while values <5 mV indicates quick aggregation of particles. Values in between the above two limits usually indicate short term stability. Other sought after properties of polymeric nanoparticles are drug loading, drug encapsulation efficiency, drug release behaviour and *in vitro* stability (Danhier et al., 2012; Lu et al., 2011; Soppimath et al., 2001).

The motivation behind this paper is to determine whether drug release patterns of polymeric nanoparticles can be related to their pore structural evolution. Although poorly investigated, this area is important because it will provide greater insight and understanding of how nanoparticles evolve over time and what mechanism(s) control(s) drug release. Moreover, the information obtained can also be fed back to scientists involved in nanoparticle syntheses allowing them to prepare nanoparticles with optimum properties, *i.e.*, desirable release profiles. Such a study therefore implies the application of a characterisation method which explores the evolution of the pore structure of polymeric nanoparticles. The experiments will also help determine if pores are generated on the outside surface or inside the particle and how they affect drug release. It will also provide a relative comparison of the nanoparticle pore size distributions (PSDs). The method investigated in this paper is NMR cryoporometry (Mitchell et al., 2008; Petrov and Furo, 2009; Strange et al., 1993; Webber, 2010).

To our knowledge, no previous workers have employed NMR cryoporometry to study PLGA nanoparticles. Earlier work mainly focussed around the use of chromatography (to determine drug content (Averineni et al., 2012)), differential scanning calorimetry (DSC) (to determine if the drug is well dispersed in the polymer matrix) (Averineni et al., 2012; Sant et al., 2005), microscopy techniques such as AFM, SEM and TEM (to determine the morphology and size of nanoparticles) (Almeria et al., 2011; Averineni et al., 2012; Avgoustakis et al., 2002; Gryparis et al., 2007; Hammady et al., 2009; Rescignano et al., 2012; Sant et al., 2005; Sawyer et al., 2011; Zhou et al., 2013; Zhu et al., 2009), DLS (to determine particle size) (Averineni et al., 2012; Rescignano et al., 2012; Sant et al., 2005), electrophoresis (to determine ζ potential) (Averineni et al., 2012; Gryparis et al., 2007), *in-vitro* and/or *in-vivo* drug release kinetic studies (Almeria et al., 2011; Averineni et al., 2012; Avgoustakis et al., 2002; Hammady et al., 2009; Sant et al., 2005; Sant et al., 2008; Sawyer et al., 2011; Zhou et al., 2013; Zhu et al., 2009) and *in-vitro* cytotoxicity studies (Averineni et al., 2012; Sawyer et al., 2011). A limited number of studies have been conducted using pore structure characterisation techniques on nanoparticles even though there are many studies related to the pore structure characterisation of micron-sized particles and mesoporous silica materials (Lee et al., 2010; Lemaire et al., 2003; Messaritaki et al., 2005; Perkins et al., 2010; Petrov et al., 2006; Ukmar et al., 2011; Zhu et al., 2009). Nitrogen gas adsorption was used to study the microporosity of pegylated nanoparticles and relate them to their drug release behaviour. The presence and arrangement of PEG polymer chains grafted on to the nanoparticle surface or internalised into the nanoparticle matrix affected the microporosity of particles and thereby the drug release kinetics (Sant et al., 2008). Earlier work by the same group provided evidence of the change in the pore size distributions and surface fractal dimensions depending on the formulation of the nanoparticles (especially their drug loading and release kinetics) (Sant et al., 2005). Another example of the application of nitrogen gas adsorption is in the structural characterisation of templated mesoporous materials (SBA-15 containing γ-Fe_2_O_3_ nanoparticles and a temperature responsive PNIPAM polymer) used as stimuli responsive drug delivery agents (Zhu et al., 2009). However, it should be noted that gas adsorption has an important limitation, in that it requires a dry sample. Drying of polymeric nanoparticles may cause changes, or even damage, to the pore structure due to the sensitivity of the polymer to humidity and high temperature. Thus, the properties measured may not be accurate, or even reflect the sample in the wetted state during use.

Thermoporosimetry using DSC has also been used to study the pore structure of porous biopolymeric (PLGA and PLLA) nanoparticles (Rescignano et al., 2012). These DSC experiments were supported by field-emission SEM images which highlighted the porous nature of nanoparticles. The Gibbs–Thomson equation (see Eq. (3.1) in Section 3) was used to estimate the pore size within these particles. These authors pointed out that the pore structure could have an important implication on drug release kinetics but did not relate structural evolution to release profile. DSC has also been used to study the drug release behaviour from albumin nanoparticles contained within a carboxylated polyurethane matrix (Martinelli et al., 2011). An investigation of the water freezing and melting, water uptake and drug release kinetics was used to purportedly determine the two main factors that contributed to controlled drug release mechanisms. Firstly, Martinelli et al. (Martinelli et al., 2011) reported burst release was due to a swelling mechanism, while controlled release was due to a diffusion dependent mechanism. They attributed the above two mechanisms to the presence of freezing and non-freezing fractions which aided the presence of a dual diffusion barrier that prolonged the drug release in their materials (Martinelli et al., 2011). However, a particular issue arises with the use of DSC as a characterisation method. This is because it simply measures the amount of heat released or absorbed by a sample as the temperature is varied. This heat flow need not originate just from the liquid in the pores of the material but also from changes in the polymer itself which cannot be independently distinguished. In contrast, the NMR method used in this paper monitors liquid mobility, and, if necessary, can also distinguish between different chemical species.

Apart from the disadvantages highlighted for the two methods previously used, earlier workers have also not related different types of drug release characteristics to pore structural properties. In the following sections, details will be given regarding the synthesis of different sets of nanoparticles, their characteristic properties, and the methodology used to determine their respective drug release profiles. Details regarding the characterisation method of interest, NMR cryoporometry and its theory are also provided. In particular, the potential issue for cryoporometry, with more realistic probe fluids, of salt exclusion will be considered to determine if it is significant for interpreting the data for the system studied here. Cryoporometry, using artificial cerebrospinal fluid (aCSF) as the probe fluid, will be performed on a model material. The results obtained for each of the nanoparticles will be discussed individually. This will link pore structural evolution to drug release experiments.

## 2 Materials and methods

### 2.1 Materials

Poly(lactide-co-glicolide) (PLGA) polymers with a 1:1 co-polymerization ratio (ester ended RG504, MW ∼38 k–54 kDa, acid ended RG504H, MW ∼38 k–54 kDa, and RG502H, MW ∼7 k–17 kDa), poly(lactic acid) (PLA R203H, MW ∼18 k–24 kDa), dichloromethane (DCM), ethyl acetate (EA) and Nile Red were purchased from Sigma–Aldrich, UK. Polyvinyl alcohol (PVA, MW ∼22 kDa) was supplied by MP Biomedicals, USA. Carboplatin solution (10 mg/ml) was obtained from Accord Healthcare Limited, UK. aCSF was purchased from R&D systems Europe Ltd., UK. The composition of aCSF was disodium hydrogen phosphate 19.1 g, magnesium sulphate 30 g, potassium chloride 30 g, sodium bicarbonate 190 g, sodium chloride 730 g, hydrochloric acid qs to between pH 7.1–7.3, and fresh distilled water to make up to 100 l. The ionic strength of the aCSF was 0.145 mol l^−1^. All reagents were analytical grade or above and used as received, unless otherwise stated. The different formulations of each batch of nanoparticles are shown in Table 2. Also shown in Table 2 are the percentages of final product yields to the initial polymer input. The observed weight loss was mainly due to the removal of the excess surfactant and large particles pellets in the centrifuge purification process. Batch D was DegraFlurex PGLA (75:25) 200 nm nanoparticles containing Carboplatin, sourced commercially from Phosphorex Inc., Hopkinton, MA.

### 2.2 Nanoparticle (NP) synthesis

#### 2.2.1 PLGA nanoparticles (samples from batches A, B, and C)

PLGA nanoparticles loaded with carboplatin were prepared using the double-emulsion method (W_1_/O_1_/W_2_). 60 mg PLGA (RG504 and RG502H) was dissolved in 2 ml DCM/EA (2:8 V/V, O_1_ phase) and 0.7 ml aqueous solution of carboplatin (10 mg/ml, W_1_ phase) was emulsified in the PLGA solution using a micro-tip probe sonicator (Model VC 600, Sonics & materials Inc., UK), set at level 4 for 3 min. Level 4 corresponded to 20 kHz at 45 W cm^−2^, and had been calibrated previously (Skinner and Price, 2012). The primary (W_1_/O_1_) emulsion was transferred into 40 ml of 2.5% PVA solution (W_2_ phase) and the mixture was probe sonicated at level 4 for 5 min. The W_1_/O_1_/W_2_ emulsion was kept open and agitated by a magnetic stirrer overnight at room temperature to remove the organic solvent. In order to obtain particles with the desired diameter, the particle solution was treated by centrifugation (Centrifuge 5804R, Eppendorf, UK) at 9000 rpm for 15 min which caused the large particles to form a pellet, while the smaller particles remained in the supernatant. The pellet of large nanoparticles was removed while nanoparticles in the supernatant were collected and washed by ultracentrifugation (40,000 rpm for 20 min, Motor type 70Ti/70.1Ti, L-80 ultracentrifuge, Beckman Coulter, UK). This pellet of nanoparticles was re-suspended in water, freeze-dried and stored at 253 K for further usage. To prepare fluorescent particles (batch A), the same procedure was followed, but Nile Red was first dissolved in DCM to form a 1 mM solution, and 60 μl of this Nile Red solution was added to the O_1_ phase. Nanoparticles were synthesised with Nile Red to see if the presence of this compound influenced the structural evolution of the nanoparticles during incubation compared to carboplatin.

#### 2.2.2 PLGA–PLA nanoparticles (samples from batch E)

PLGA/PLA nanoparticles were prepared by O/W emulsion solvent evaporation techniques. PLGA (RG504H) was dissolved in 1 ml organic solvent DCM/EA (2:8 V/V) to form ‘solution A’ while PLA (R203H) was dissolved in 1 ml organic solvent DCM/EA (2:8 V/V) to form ‘solution B’. Both the A and B solution concentrations were 10% (W/V). PLGA/PLA weight ratios ranged from 1 to 3 (W/W). 10 mg carboplatin powder was added to small amounts of the DCM/EA solution, and sonicated using an ultrasonic probe for 5 min to break down the drug powders into small particles prior to the addition of ‘solution A’. The PLGA ‘solution A’ and carboplatin particles were sonicated for 2 min before adding the PLA ‘solution B’. Then the mixture of two polymeric solutions with drug particles was sonicated for another 2 min. The resultant solution was added drop wise into 40 ml of PVA aqueous solution (2.5%, W/V) followed by another 5 min sonication to create an O/W emulsion. All the sonications were performed using a micro-tip probe sonicator (Model VC 600, Sonics & materials Inc., UK) set at level 4. The emulsion was stirred overnight to allow the evaporation of organic solvent and hardening of the particles. The particles in the final solution were purified, washed and collected in the same process as mentioned in Section 2.2.1.

### 2.3 Particle size and ζ-potential characterisation

Freeze-dried nanoparticles were completely re-suspended in pure water (pH 7) by agitation in an ultrasonic bath (Fisherbrand FB 11020) for 15 min. A Zetasizer nano from Malvern Instruments using light from a He–Ne laser source (*λ* = 633 nm) was used for dynamic light scattering (DLS) measurements to determine particle size and polydispersity. All experiments were performed at 298 K at a scattering angle of 173° to the incident beam, with an assumed refractive index ratio of 1.59 and viscosity of 0.89 cP. The correlation decay functions were analysed by the cumulant method to obtain an average hydrodynamic particle size and polydispersity. The intensity-weighted mean value presented is the mean of three measurements. ζ-potential was also determined with the Zetasizer nano ZS. By measuring the electrophoretic mobility, the ζ-potential can be calculated using the Henry equation within the Smoluchowsky approximation.

### 2.4 *In vitro* carboplatin release study

The ultimate intended use of the nanoparticles would be as drug delivery vehicles to the brain and so artificial cerebrospinal fluid (aCSF) solution was used for the drug release studies. Nanoparticles (5 mg) were suspended in 0.5 ml aCSF solution and placed in a centrifuge filter tube (MWCO ∼10 kDa), and subsequently in an oven at 310 K. Samples were centrifuged at 11,000 rpm for 15 min and all aCSF solution was collected and replaced with a fresh aliquot at predetermined intervals. Finally, the remaining particles were then collected, dissolved in 0.6 ml DCM and vortexed for 3 min. 1 ml aCSF was added to the solution and the mixed solution was then vortexed for 3 min to extract carboplatin into aCSF solution. This solution was then centrifuged at 11,000 rpm for 15 min, and the upper aqueous solution containing the extracted carboplatin was removed by using a pipette and collected before fresh aCSF solution was replaced onto the DCM solution. The whole extraction process was repeated five times to make sure that any remaining carboplatin in the particles was fully extracted. The carboplatin concentration in the collected solutions was measured by Inductively Coupled Plasma Mass Spectrometry (Thermo Fisher Scientific XSeries 2 ICP-MS) at the Southampton Oceanography Center, UK.

### 2.5 NMR cryoporometry

NMR cryoporometry experiments using aCSF solution were performed on each sample of nanoparticles. The samples were first immersed in aCSF solution within a pipette tip. The tip was then sealed with parafilm and introduced into an NMR tube. A tissue wetted with aCSF solution was placed at the top of the NMR tube and then sealed with a cap to ensure the environment was always saturated. This sealed NMR tube was then incubated at 309.7 K. The NMR cryoporometry or freeze–melt experiments were performed periodically using a Bruker Avance (III) spectrometer with static field strength of 14.75 T, corresponding to a resonance frequency of 600 MHz for ^1^H nuclei. Proton intensities were obtained at different temperatures using a 1D spin echo sequence: T-90x-180y-echo, where the echo time was 1250 μs and the relaxation delay time was 5 s. The probe used was a ‘smart probe plus’, which was connected to a BCU II chiller system. The first spectrum was obtained at 278.5 K, following which the sample was cooled to 217.8 K, since it was established that there was no liquid-state signal at this temperature. The system was tuned at this temperature. Thereafter, a melting cycle was initiated using an automation program with 0.5 K increments from 218 K to 278 K, where tuning was also carried out at each temperature. The equilibration time at each temperature was set at 540 s. In earlier work (Perkins et al., 2010), it was established that this equilibration time was sufficiently long that the results do not depend on equilibration time. All experiments on a particular sample were begun and ended at identical conditions. The spin echo spectrum at 278.5 K was used as the reference spectrum for each run. Further, the receiver gain value was not changed between the different runs, so the intensities were on the same scale.

### 2.6 Transmission electron microscopy (TEM), environmental SEM (ESEM) and differential scanning calorimetry (DSC)

Morphological evaluation of the nanoparticles was performed using TEM (JEOL JEM 1200 EXII). A drop of NP suspension was placed on a Formvar (poly(vinylformal))/carbon supported 400 mesh grid, excess liquid was removed with a filter paper, and the grid was dried in air at room temperature followed by negative staining with phosphotungstic acid solution (3% W/V). TEM images were calibrated using SIRA gratings 2160 (462 nm) lines per millimeter and catalase crystals for high magnification (8.75/6.85 nm spacing) in the unit cell.

The morphology of the nanoparticle samples were also studied using a PC controlled Philips XL 30 FEG ESEM. This instrument can be used in the wet water mode or as a conventional SEM. The ESEM images were obtained by taking a representative amount of the nanoparticle sample and placing them on the sample holder (Batch A) or by dispersing them in water and introducing the same to the sample holder (Batch C). Images of Batch A were obtained by gradually increasing the humidity from 25% to ∼90% and evacuating back to 25% while batch C image was obtained in the high vacuum mode.

A differential scanning calorimeter (Q10 DSC, TA Instruments), equipped with a cooling unit (RCS, Refrigerated Cooling System) for controlled cooling, was used for evaluation of glass transition temperature. A constant furnace atmosphere was maintained with an in-house nitrogen purge.

## 3 Theory

NMR cryoporometry is a technique based on the principle of studying the melting point or freezing point depression of liquids confined in porous materials. The phase change of liquids can be related to size of the pores in which it is confined by the Gibbs–Thomson equation:

(3.1) $$\Delta T_{m}=T_{m}^{\infty }-T_{m}(x)=\frac{4\sigma_{\text{cl}}T_{m}^{\infty }}{x\Delta H_{f}\rho_{s}}\cos(\phi )$$

where $\Delta T_{m}$ is the melting point depression; $T_{m}^{\infty }$ is the normal bulk melting point of the solid crystal; *T*_m_(*x*) is the melting point of crystals of diameter *x*; $\sigma_{cl}$ is the surface energy of the crystalline-liquid interface; $\Delta H_{f}$ is the enthalpy of fusion of the material; *ρ*_s_ is the density of the solid, and ϕ is solid crystal-liquid contact angle (often assumed to be 180°). The above equation can be simplified to:

(3.2) $$\Delta T_{m}=T_{m}^{\infty }-T_{m}(x)=\frac{k_{\text{GT}}}{x}$$

where *k*_GT_ is the Gibbs–Thomson coefficient. *k*_GT_ can be obtained when melting point depressions are plotted against the inverse of the crystal size. It is a characteristic of the confined liquid and sets limit on the pore size measured. It is well known that solids, liquids and gases have different NMR spin relaxation times. NMR cryoporometry exploits the difference in the *T*_2_ spin relaxation times between solids and liquids confined within porous materials such that only the signal from the liquid phase is detected. The transition from liquid-to-solid, or solid-to-liquid, can thus be monitored by the decay or rise in NMR signal strength, respectively. This, along with accurate temperature control to determine the pore size of the transition, can be used to characterise the pore structure (Mitchell et al., 2008; Petrov and Furo, 2009; Strange et al., 1993; Webber, 2010).

However, due to the likely inappropriateness of the bulk values, and the difficulty in predicting, *a priori*, the values of the physical parameters in the Gibbs–Thomson equation for highly confined geometries, the value of *k*_GT_ is usually obtained by calibration of Eq. (3.2) using a porous solid with a previously known pore size obtained by independent means. However, there are no obvious candidates for model polymer materials with controlled regular pore size that could be used to calibrate the Gibbs–Thomson equation in a fashion analogous to use of MCM-41 or SBA-15 for silicas. Further, in this work, the key aim is to monitor the evolution of the same void space over time and thus only relative indications of pore size are essential. Hence, in the following discussions, comparisons of the pore size changes over time are made in terms of the proxy melting temperature. However, where an indicatory pore size is of use, a typical value for *k*_GT_ of 50 nm K (for oxide materials, based on diameter) (Mitchell et al., 2008; Petrov and Furo, 2009; Strange et al., 1993; Webber, 2010) will be employed to obtain the pore sizes mentioned in the following text. As will be seen below, this value is very close to that for aCSF in a silica model material.

Artificial cerebrospinal fluid contains a mixture of salts, and thus salt exclusion effects during freezing and melting might be important. In previous work (Perkins et al., 2010), a comparison of freezing curves for sodium chloride soaked PLGA samples and those in pure water showed that no salt exclusion effects were evident. Progressive freezing of the fluid within the void space might conceivably cause salt exclusion that would be expected to progressively generate more concentrated brine in ever smaller molten regions that would thus have ever more depressed melting points. Due to intra-batch/inter-sample variability in amorphous porous media, this process might be expected to have a random character. It has been found (Perkins et al., 2010) that, for a nanoporous sol–gel silica material, the position of the nanopore network melting curve was depressed by 2 K for experiments with 5% W/V sodium chloride solution compared to analogous experiments with pure water. However, within experimental error, the curves could be superposed once the temperatures for data-points in the water curve had all been reduced by 2 K. Since, if occurring, the aforementioned salt exclusion effects might be expected to broaden the distribution of freezing and melting temperatures, compared to that for pure water, this finding suggested that salt exclusion effects on the shape of the distribution were negligible. Previous work (Perkins et al., 2010) has considered salt exclusion using 5% W/V sodium chloride solution as the probe fluid. Since the experiments on PLGA were run over an extended period of time (Perkins et al., 2010), effects arising from the progressive concentration, or dilution, of the (bulk and pore) salt solution due to liquid evaporation, or interaction of the salt with the polymer, respectively, might be anticipated. However, no systematic change in the melting point for the bulk solution with time was seen in the experiments, and thus the effects of evaporation (from the sealed container) and long-term salt–polymer interaction were considered negligible. The lack of change of bulk melting point indicates no significant salt–polymer interaction for the following reasons. If there is a salt–polymer interaction then the ions are likely to adsorb on the polymer, which being micro-/nano-particulate and having nanopores will have a large surface area for this to occur, if it were to do so to a large extent. This would mean that the bulk liquid surrounding the nanoparticles would be depleted of ions, and thus the effect of the ions on the bulk melting point would be reduced, and it would be expected that the bulk melting point would be less depressed. This effect is not seen and thus there cannot be any significant ion-polymer interaction. It has also been checked that the frequency of cryoporometry experiments used does not, itself, alter the trajectory of the pore structure evolution in PLGA samples.

## 4 Results

### 4.1 Cryoporometry of model silica material

**Fig. 1:**
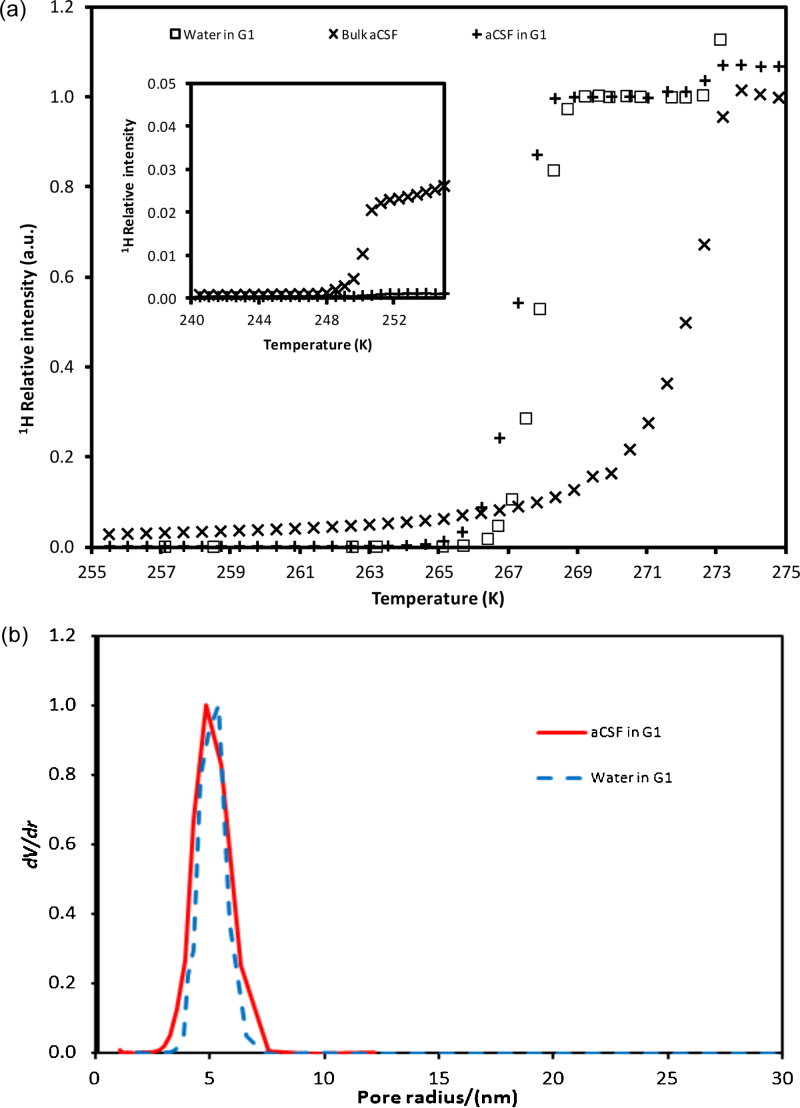
(a) NMR cryoporometry melting curve data for bulk aCSF solution, and for aCSF imbibed in silica G1, and also for pure water in G1. The inset shows the same data curves at low temperatures. (b) Differential pore size distributions derived from melting curves for pure water and aCSF imbibed in samples from batch G1.

The model material used was a sol–gel silica sphere, denoted G1, which has been fully described and characterised using gas sorption and mercury porosimetry previously (Perkins et al., 2010). The pore size distribution obtained from a calibrated version of the Washburn equation was found to have a modal pore size of ∼10 nm. Cryoporometry melting curves were obtained for G1 using either aCSF or pure water as the probe fluid, and the results for both are shown in Fig. 1(a). Also included in Fig. 1(a) is the melting curve for bulk aCSF alone. For samples with G1, the small step at high temperature (∼273 K) is attributed to the melting of the film of bulk liquid surrounding the pellets, while the larger step at lower temperature is attributed to melting of intra-particle fluid. From Fig. 1(a), it can be seen that melting within G1 is depressed below the bulk melting point for water of 273.15 K. The melting point depression of aCSF within G1 is ∼5 K, which, in conjunction with the known modal pore size for G1, suggests a value of *k*_GT_ of 50 nm K is probably reasonably appropriate for aCSF. It is also noted that the form of the melting curve for aCSF within G1 is very similar to that for pure water, though just shifted slightly lower in temperature. However, the melting curve step for aCSF within G1 is much sharper than the melting curve step for bulk aCSF. At the lowest temperatures (below 247 K) the melting curve for bulk aCSF is relatively flat with little signal, but at about ∼250–251 K there is a pronounced step up in intensity, followed by a long slow climb in intensity with increasing temperature, before increasing much more sharply in intensity above 270 K. The melting curve for aCSF within G1 does not have this same long tail, or the same pronounced step at ∼250–251 K. The ∼250–251 K step for the bulk aCSF sample is attributed to the melting of the pocket of high salt concentration solution produced by salt exclusion. The very slight bump in the curve for aCSF and G1 at the same temperature may be due to the same salt exclusion effect within the small amount of bulk aCSF present outside the pellets themselves, the presence of which is indicated by the small step at ∼273 K for the pellet samples. In Fig. 1(b) it can be seen that the superposed differential pore size distributions derived from the cryoporometry melting curves for water and aCSF, respectively, within G1 are very similar in form.

### 4.2 Nanoparticle properties

The characteristic properties of the different nanoparticulate formulations are given in Table 3. The particle size for the different batches was between 145 and 195 nm. Batch D, the commercial sample, was found to have the smallest particle size, while batch E had the largest particle size. The polydispersity for all batches was less than 0.2 indicating a narrow size distribution. The zeta potential of PLGA nanoparticles can be affected by the polymer end group. In Table 3, the zeta potential is −23 mV for the ester-terminated, and −33 mV for the COOH-terminated, PLGA-NPs, which is close to values previously reported by Ghotbi et al. (Ghotbi et al., 2011). The zeta potential for pure PLGA nanoparticles prepared without any PVA in a neutral environment is about −45 mV. This high negative charge is due to the presence of uncapped end carboxyl groups of the polymer at the particle surface. In our study, the nanoparticles were all prepared with the emulsifier polyvinyl alcohol (PVA), a fraction of PVA remains associated with the nanoparticle, despite repeated washing, since PVA forms an interconnected network with the polymer at the interface. The PVA layer at the nanoparticle surface probably shields the PLGA surface charge; and particles with less shielding should be expected to have larger absolute zeta potential value. According to Sahoo et al. (Sahoo et al., 2002), the PVA residual in our nanoparticles should be less than 0.5%. DSC data (not shown) was also obtained for the nanoparticles, with a ramp rate of 10 K min^−1^ from 193 to 523 K. In addition to a sharp water peak at 373 K, the glass transition was also observed, which occurred at 316 K for batch A, for example, which is the same as that observed for similar PLGA particles (Passerini and Craig, 2001).

### 4.3 Batch A – ESEM and NMR cryoporometry with aCSF

**Fig. 2:**
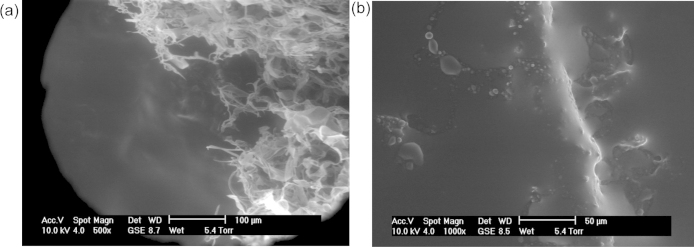
ESEM (a and b) images of aggregates of polymer nanoparticles, from batch A. Images (a) and (b) were obtained after exposing the sample to 100% RH for 124 and 128 min respectively. The time quoted is the cumulative exposure time. Image (a) shows the suspension of nanoparticles, and Image (b) shows the gel into which the aggregates changes.

Batches A and B were identical except that batch A contained Nile Red, while batch B contained carboplatin. The pore structural evolution of nanoparticles from batch A following exposure to water vapour, or immersion in aCSF solution, was studied using microscopy techniques or NMR cryoporometry respectively. These two methods enabled the study the nanoparticle structure over both very short and long evolutionary time scales, *i.e.* of the order of minutes and days. Nanoparticles from batch A were exposed to increasing levels of relative humidity (RH), from ∼5% to 100%, to study the interaction of the sample with water vapour. At ∼100% RH, a gel or paste formation was observed. Water vapour condensed in particular isolated regions of the sample, and then began flooding the sample, leading to the formation of a suspension, as seen in Fig. 2(a). Fig. 2(a) was obtained after exposing the same to water vapour for ∼2 h. A few minutes later, a thick gel had formed, as seen in Fig. 2(b). Upon drying the sample to ∼48% RH and then to 21% RH, the paste remained visible.

**Fig. 3:**
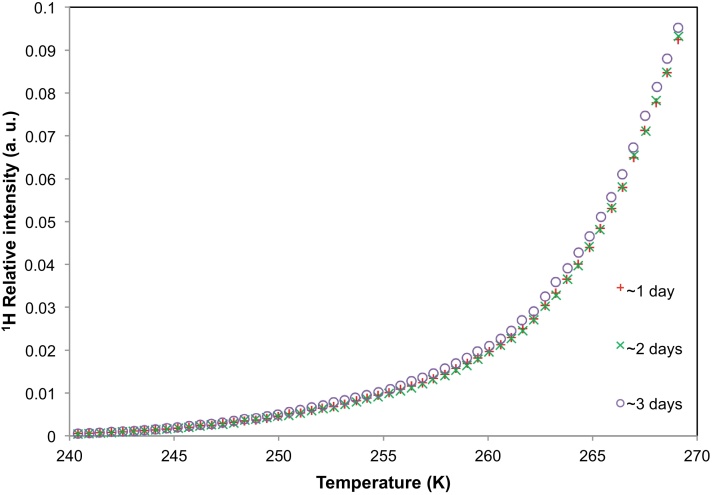
NMR cryoporometry data for polymer nanoparticles from batch A immersed in aCSF. The melting profiles of the aCSF solution containing the nanoparticles were obtained after ∼1 day, ∼2 days and ∼3 days of incubation.

The ESEM images provided a qualitative understanding of the interaction between water vapour and the nanoparticle aggregate structure during short time intervals. However, the NMR cryoporometry technique was used to study the evolution of the particle nanoscale structure, following immersion in aCSF, over longer time intervals. Evolution of the nanoparticle structure would be evident from any visible shifts in the melting profile of the probe fluid. As longer time scales were of interest, the melting profiles of aCSF solution containing nanoparticles belonging to batch A were obtained periodically after 1, 2 and 3 day(s) of incubation at 309.7 K. Fig. 3 presents the melting profiles of aCSF solution containing nanoparticles of batch A. The melting curves obtained after ∼1 day, ∼2 days, and ∼3 days of incubation showed no significant difference from each other.

### 4.4 Batch B – TEM and NMR cryoporometry with aCSF

**Fig. 4:**
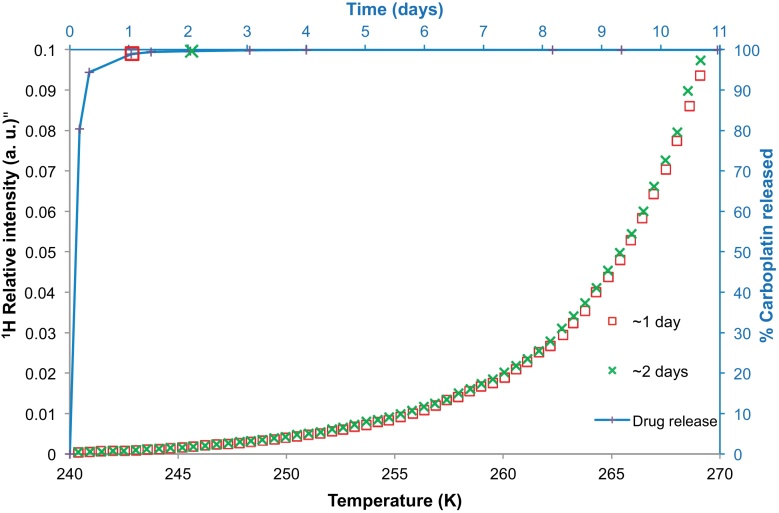
NMR cryoporometry data (chains of symbols) for polymer nanoparticles belonging to batch B immersed in aCSF. The melting profiles of the aCSF solution containing the nanoparticles were obtained after ∼1 day, and ∼2 days of incubation. The percentage carboplatin released from the nanoparticles of batch B has been plotted (with line shown to guide the eye) against time on the secondary axes, with melting curves taken at the times indicated by the relevant symbol.

NMR cryoporometry was used to study the pore structural evolution of polymer nanoparticles from batch B immersed in aCSF. Fig. 4 presents the melting profile of aCSF solution containing the nanoparticles from batch B. The melting profiles were obtained after ∼1 and ∼2 days of incubation. Fig. 4 also shows the carboplatin drug release profile for these nanoparticles. The individual symbols situated at various points along the release profile correspond to the times at which the melting profiles (displayed with same symbol shape) were obtained. It was found that ∼98.9% of the drug was released in 24 h and over the next 48 h there was only a ∼1% increase in the drug released. The melting profiles of the solution containing nanoparticles after ∼1 and ∼2 days of incubation were found to be very similar showing very little increase in pore volume over the period investigated. The data obtained after 2 days, shown in Fig. 4, are located above the data obtained after 1 day of incubation. It was also noted that the step present in the melting curve for the bulk solution at a temperature of ∼250–1 K was absent in the data for the solution containing nanoparticles (similar to batch A seen previously). Instead the melting profile was smooth and broad over the entire temperature range.

**Fig. 5:**
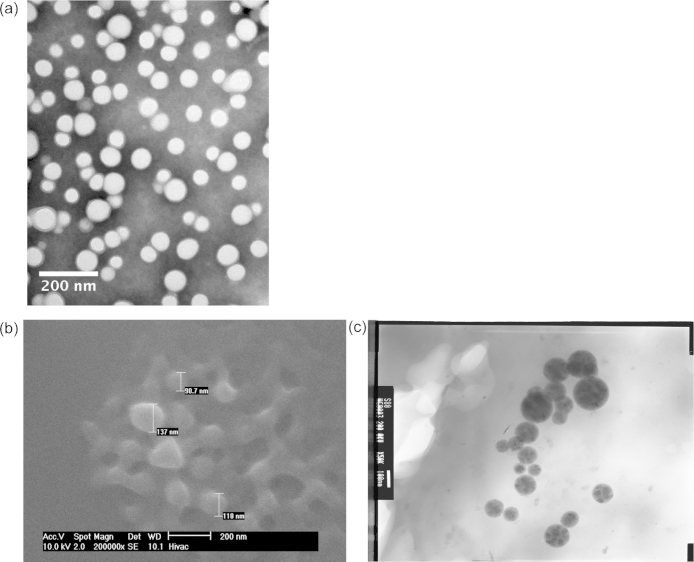
(a) TEM images of nanoparticles from batch B negatively stained with phosphotungstic acid. ESEM (b) and TEM (c) of polymer nanoparticles from batch C. The images were obtained after preparing a suspension of nanoparticles in water. The images show the size and morphology of the nanoparticles.

It is also noted that the melting profiles obtained for batches A and B are very similar in shape over the time intervals studied. Fig. 5(a) shows a TEM image of nanoparticles from batch B. It can be seen that they have a round morphology.

### 4.5 Batch C – ESEM, TEM and NMR cryoporometry with aCSF

**Fig. 6:**
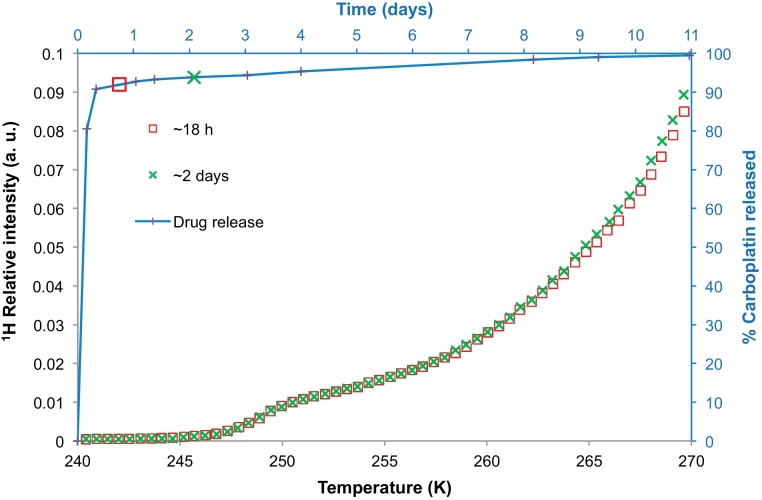
NMR cryoporometry data (symbols) for polymer nanoparticles from batch C immersed in aCSF. The melting profiles of the aCSF solution containing the nanoparticles were obtained after ∼18 h and ∼2 days of incubation. The percentage carboplatin released from the nanoparticles has been plotted (with line shown to guide the eye) against time on the secondary axes. The lone symbols also located along the line, matching those from the melting curves, indicate when those melting profiles were obtained.

Batch C was studied using ESEM, TEM and NMR cryoporometry. The ESEM and TEM images were obtained after dispersing the sample in water and then placing them on a grid. They clearly showed the presence of spherical nanoparticles, as seen in Fig. 5(b and c). These nanoparticles are of the order of ∼100 nm in size. The melting curves of aCSF solution containing nanoparticles from batch C, and the nanoparticle drug release profile are plotted in Fig. 6. The drug release profile shows a pulse release of ∼90% of the drug within the first half-day, and thereafter a long slight tail. The melting curves of the aCSF solution containing nanoparticles from batch C were obtained after ∼18 h and ∼2 days. As seen earlier for batch A and batch B, the melting curves were similar after ∼18 h and ∼2 days. However, unlike the smooth melting curves obtained for samples from batches B and A, the melting curves of sample C had the presence of a steeper melting step. The time points at which each melting profile was obtained can be seen in the drug release profile, indicated by each lone symbol corresponding to the appropriate melting profile. As evident from the drug release data, there is very little change in shape of the drug release profile during the time interval investigated. This also correlated with the little change in melting profiles of the aCSF solution in the same time period.

### 4.6 Batch D – NMR cryoporometry with aCSF

**Fig. 7:**
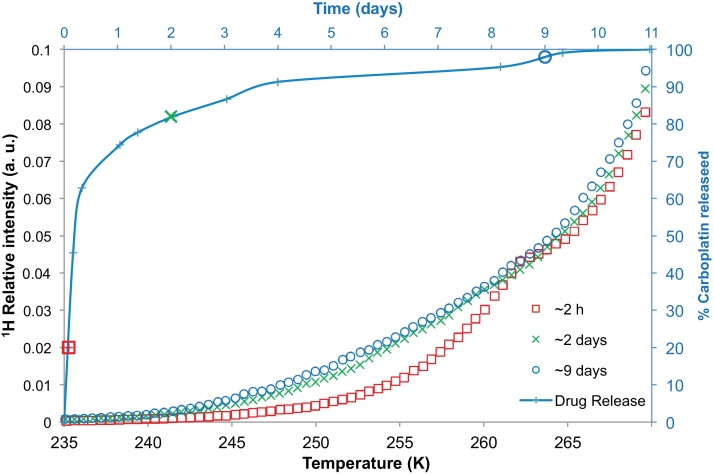
NMR cryoporometry data (symbols) for bulk aCSF solution, and for polymer nanoparticles from batch D immersed in aCSF at different time periods, namely ∼2 h, ∼2 days and ∼9 days. The percentage carboplatin released has been plotted (line) against time on the secondary axes.

The melting curves of aCSF solution containing nanoparticles from batch D, and their drug release profile are plotted in Fig. 7. It was found that nanoparticles from batch D exhibited a spontaneous or burst release of ∼60% of its drug content within the first 12 h. After this initial burst release (within half a day), the remaining ∼40% of the drug was gradually released over ∼10.5 days. A declining release rate was noted in the drug release profile tail at later times, unlike the burst release samples seen earlier. The cryoporometry melting curves were obtained after ∼2 h, ∼2 days and ∼9 days of incubation. A roughly three times increase in the volume of small to medium sized (∼2–4 nm, corresponding to ∼248–260 K) pores was evident in the melting curves when comparing the results obtained following 2 h of incubation, and following ∼2 days of incubation. This time period corresponded to a significant jump in the amount of drug released from the nanoparticles (∼62% drug released). At later times, it was found that there was little change in the melting profile which corresponded to the lesser amount of drug released over that period (∼16% drug released).

### 4.7 Batch E – ESEM and NMR cryoporometry with aCSF

**Fig. 8:**
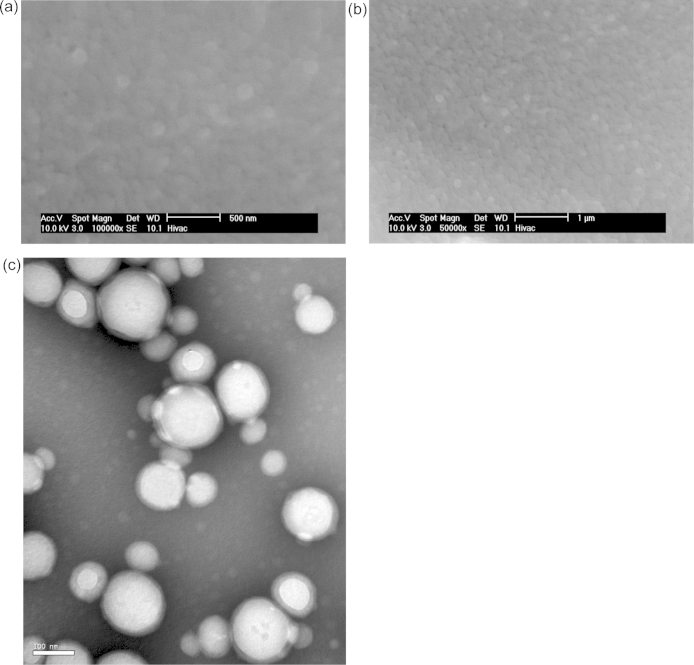
ESEM images of spherical polymer nanoparticles from batch E in the native state. (b) is a zoomed out version of (a). (c) TEM image of nanoparticles from batch E negatively stained with phosphotungstic acid. The images show the size and morphology of the nanoparticles.

**Fig. 9:**
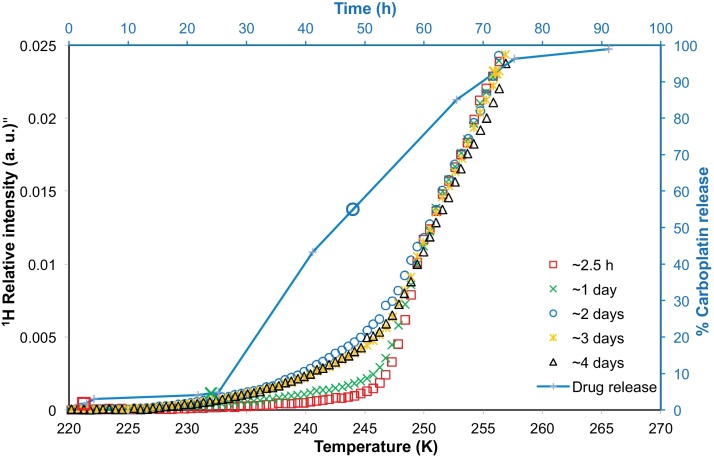
NMR cryoporometry data (chains of symbols) for polymer nanoparticles from batch E immersed in aCSF. The melting profiles of the aCSF solution containing the nanoparticles were obtained after ∼2.5 h, ∼ 1 day, ∼2 days, ∼3 days, ∼4 days of incubation. The percentage carboplatin released from batch E has been plotted (solid line shown to guide the eye) against time on the secondary axes, with symbols to indicate the times when melting profiles were obtained.

Batch E comprised spherical nanoparticles (Fig. 8) that were ≥100 nm in size. As can be seen from the drug release profile given in Fig. 9, it was observed that nanoparticles from batch E provided a very small initial burst, and then very little drug release during the next 24 h. Thereafter there was a period of significant sustained drug release lasting ∼2 days, before the release finally tailed off and reached a plateau. From a consideration of the cryoporometry melting curves for nanoparticles from batch E (Fig. 9), it was found that that the melting curves remained very similar over the period of ∼2.5 h to 1 day, with, maybe, a very slight rise in nanopore volume. However, between 1 day and 2 days, when there was a significant change in amount of drug released (∼24%), there was also a significant shift in the melting curve (Fig. 9). The shapes of the melting curves suggest that the volume of pores of sizes ∼1–2 nm (corresponding to ∼230–248 K) increased by about five times between 1 and 2 days. Following this period of change, the melting curves obtained over ∼2, ∼3 and ∼4 days were very similar. The drug release profile after 24 h was fitted to an exponential growth phenomenological mathematical model. The model fitted the data reasonably well (the coefficient of determination of the fit was 0.972) and the time constant for the function was 0.05 h^−1^.

## 5 Discussion

The absence of a pronounced, low temperature (∼250–251 K) step, similar to that observed for bulk aCSF, in the cryoporometry data (see Fig. 1(a)) for melting of aCSF within the model silica material suggested that a similar salt exclusion effect was not occurring within the porous material as occurred for the bulk-only sample. This is further confirmed by the finding that the forms of the intra-particle melting curve, and pore size distribution derived from it, for aCSF in G1 are very similar to the corresponding results for pure water in G1 (Fig. 1(b)). If salt exclusion affected the melting of aCSF imbibed in G1, as observed for the bulk aCSF sample, then the data for aCSF in G1 would be expected to be asymmetric with a long tail to low temperature (small pores). Hence, it was concluded that salt exclusion effects were not important for interpreting cryoporometry melting curves for aCSF in mesoporous solids.

It was found that the five different batches of nanoparticles studied here gave rise, between them, to three different forms of drug release profiles. The reasons for the different forms of the drug release profiles can be deduced from the complementary data obtained from NMR and EM. Both the experimental methods provided useful information on the interaction between water or aCSF solution and the nanoparticle structure. The structural evolution of nanoparticles allows water to penetrate the structure, which can be detected by NMR. The melting point of that water, in turn, reveals the size of the pores in which it resides. The presence, or absence, of these pores, and the ability of water to penetrate the structure, or not, depends on how the structure of the nanoparticles evolves.

Evidence for the presence of a correlation between the evolution of the nanoparticle pore structure and the drug release characteristics was established. Importantly, the microscopy technique provided an understanding of how water vapour interacted with the nanoparticle aggregate structure over short time scales (∼minutes or ∼hours), while the NMR cryoporometry technique revealed nanoparticle pore structural information over longer time scales. The time scales investigated varied from a few hours to a few days. Where the NMR data for a particular batch exhibited a significant increase in ^1^H NMR intensities over a particular range of temperatures, such a shift was attributed to the development, and evolution, of pores. The different batches were chosen as they exhibited different release characteristics, *i.e.*, burst (A, B, C), controlled (D, E) and delayed (D, E) release characteristics.

The steep melting step in the melting curve data for the bulk aCSF (see Fig. 1, 250–251 K) is probably due to salt exclusion effects, whereby the salts in the aCSF are steadily excluded into remaining liquid until the final highly concentrated salt solution finally solidifies, and, thence, also melts at low temperature compared to bulk. However, the shapes of the melting curves for aCSF imbibed within nanoparticle samples were very different to that for bulk aCSF, and this finding, together with those from aforementioned studies (Perkins et al., 2010) of potential salt exclusion effects for solutions imbibed within nanoporous media, suggest that salt exclusion does not contribute significantly to the results for the nanoparticle samples studied here.

The cryoporometry melting curves for aCSF imbibed within nanoparticles from batches A, B, and C all exhibited very little change over the time-scales measured. The absence of any shift in the aCSF solution melting temperatures for batches A, B and C indicated the absence of any pore structural change over the same time scales. The marked step in the melting curves for batch C around 250 K, that is not present in the melting curves for batches A and B, suggests a network of pores with a relatively narrow range of sizes, and could arise for a reason related to the fact that batch C is composed of polymer of lower molecular weight than batches A and B. In all of these batches, the vast majority of the drug (≳90%) had already been released in the first few hours of incubation. Such a burst release of the drug could possibly be due to the washing away of the drug from the surface of the nanoparticle itself. Thus, a relation was found to exist between the drug release profile and the melting profiles of batches A and B obtained at different times. Little change or shift in the melting profiles of the aCSF solution agreed with the little change in the drug amounts released. Furthermore, microscopy studies showed a rapid gel or paste formation upon exposure to ∼100% RH in batch A demonstrating the rapid response of the nanoparticle aggregate to moisture, which would facilitate the burst release of a drug. The slightly lower initial burst release for batch C (∼90% of drug), relative to batch B (∼95–99%), may be because the acid end group of the polymer used to make batch C may have had a slightly greater affinity for the drug, leading to a smaller initial burst release. The remaining 10% of the drug in batch C may be retained by the end group and thus slowly released over ∼0.5–9 days.

Unlike batches B and C, which showed predominantly burst release characteristics, batch D had an initial period that exhibited partial burst release of up to ∼60% of the drug, but this was then followed by a second period that showed substantial delayed release in a long tail. The aCSF solution melting curve obtained after ∼2 days of incubation showed greater ^1^H NMR intensity relative to that obtained earlier, after only ∼2 h of incubation. This indicated swelling and pore volume generation within the nanoparticle structure had occurred between the initial pulse release period, and the onset of the delayed tail. It is noted that this change took place over a time period when ∼60% of the drug content was released. The remaining drug content was released in a delayed manner with very little evolution in the pore structure. The absence of both any structural change and any substantial delayed release period for batches B and C, but the association, observed for batch D, between the period of the changeover from pulse to delayed release with a concomitant structural change, suggests that the mechanisms of drug release for these two periods are different, with the delayed release being associated with a more open pore structure. It can thus be inferred that release in the second period is controlled by the migration of drug through the evolving pore structure.

The behaviour of Batch E was distinctly different again from that of the other nanoparticles. It was characterised by an initial time period within which there was very little change in the nanoparticle pore structure and very little drug release. This time period was nearly 24 h. Thereafter, the drug amount released increased by a substantial amount, and that release occurred in a controlled, or sustained, manner over the following two days. It was only during the early part of this second period that the nanoparticle pore structure evolved substantially, as evidenced by the NMR cryoporometry data. This second period was characterised by the generation of accessible nanoporous void volume ∼4–5 times larger than previously present. Most of the pores were in the small size range (∼1–2 nm). At later stages, when the amount of drug released approached 90%, there was very little change in the form of the melting curve from that obtained after 3 days of incubation. Hence, the observed evolution of the pore structure is mostly confined to the period ∼1–2 days, associated with the onset of sustained release. Thereafter, the structure generally stays open allowing the drug to diffuse easily out. The synthesis of batch E was similar to the polymer-alloys method used to synthesise double-walled PLA–PLGA microspheres (Lee et al., 2002; Matsumoto et al., 1997). The form of the drug release profile for batch E, with a delay followed by a steady release, was similar to that observed for double-walled microspheres (Lee et al., 2002). In the solvent evaporation process during the synthesis of these materials the polymer total weight increases above 30% so a phase separation is achieved. Small spheres of the more hydrophilic and faster degrading PLGA become entrapped within a stronger PLA coating (Lee et al., 2002). Cisplatin tends to associate with the PLGA phase (Matsumoto et al., 2005), and it might be expected that carboplatin would behave similarly. A mechanism has been proposed for the release of drug from multi-reservoir, double-walled microspheres (Matsumoto et al., 2005). Initially, only drug bound loosely to the exterior of the microsphere is released. Subsequently, erosion of the outer PLA coating occurs, forming a few micropores. Thereafter, erosion of the PLGA core occurs and the drug is released. Finally, more effective pores in the drug-free PLA layer are generated. The small size of the nanoparticles meant that the same microscopy methods used to study microparticles (Lee et al., 2002; Matsumoto et al., 1997; Matsumoto et al., 2005) could not feasibly be used for the nanoparticles. However, cryoporometry offers another means by which particle structural evolution can be monitored at the nanoscale. The cryoporometry results have shown that drug release from batch E nanoparticles is generally consistent with the aforementioned mechanism for microparticles (Matsumoto et al., 2005). Upon initial immersion in aCSF, a very small amount of drug is released from the exterior surface of the nanoparticles. There is then an induction period of ∼1 day and the slight rise in the melting curve over this period (in Fig. 9) might be associated with the erosion of some micropores in the outer PLA coating of the nanoparticles. The sudden release of drug and substantial increase in pore volume in the melting curve may be associated with the final penetration of the micropores in the PLA through to the PLGA core which is more rapidly degraded. The exponential growth form of the drug release profile, once breakthrough of the microporous passages to the core has been achieved, means that the rate of drug release declines with remaining drug concentration, which would be consistent with a diffusional mechanism, whereby the drug has to diffuse from the core through the now permeable outer barrier layer.

Hence, it has been shown that the forms of the NMR cryoporometry melting curves presented in this paper correlated strongly with forms of the drug release profile data. The work showed that drug release experiments alone cannot provide an understanding of the pore structural evolution of polymeric nanoparticles. It is only by comparison with a pore structural characterisation technique that a mechanistic understanding can be achieved. In contrast to our approach and findings, Martinelli et al. (Martinelli et al., 2011) have used DSC and drug release experiments to determine that the burst release effect is correlated with the presence of a larger freezing fraction when the sample is frozen, and arises due to swelling. On the other hand, controlled release was correlated by them with the presence of a higher non-freezing fraction during thermoporometry. A reason for the difference with our findings described here could be due to the different polymers involved in the respective studies.

## 6 Conclusion

This work has directly demonstrated that different synthesis routes for nanoparticles leads to different pathways in the evolution of the pore structure which gives rise to the different observed drug release profiles. This work has elucidated this previously unclear link between synthesis method and release profile. This has enabled theoretical proposals for the specific mechanism(s) of drug release for nanoparticles synthesised in a particular way to be tested and validated. In particular, the rationale for the design of the synthesis route for a core-coat structured nanoparticle, and the proposed mechanism for delayed drug release in such particles, have been independently validated. Hence, the cryoporometry method permits a more intelligent design of nanoparticle synthesis to achieve a particular release profile.

## Figures and Tables

**Table 1:** Properties associated with different release formulations.

Formulation	Properties
Extended release formulation	Initially produce a therapeutic effect and then continue with release which is not necessarily constant
Sustained release formulation	Drug release with kinetic profile of zero order. Therapeutic effect is maintained over a long period of time
Pulsatile release formulation	Drug release in a pulsatile manner as and when required by the body. Drug is released in accurate amounts at the right time and place
Delayed release formulations	Drug release does not coincide with the time of administration. Its therapeutic action is not extended. The presence of coatings protects the active ingredients

**Table 2:** Different formulations and yields of nanoparticles.

Batch code	Formulation	Yield/%
A	RG504-Nile Red-NP	7
B	RG504 38 k–54 kDa	37
C	7 k–17 kDa RG502H	40
D	(sourced commercially)	–
E	PLGA (RG504H): 2 PLA (R203H) NP	34

**Table 3:** Different formulations of nanoparticles and their characteristic properties.

Batch	Particle diameter (nm)	Polydispersity	ζ-potential (mv)	Carboplatin drug loading/wt%
A	190.7 ± 1.7	0.089 ± 0.013	−28.8 ± 0.5	–
B	182.8 ± 3.9	0.092 ± 0.019	−22.9 ± 0.9	0.30
C	167.7 ± 0.3	0.093 ± 0.021	−32.8 ± 1.0	0.17
D^a^	146.2 ± 1.5	0.115 ± 0.032	−25.2 ± 0.5	0.0072
E	193.5 ± 0.9	0.097 ± 0.010	−26.5 ± 0.4	1.3

a Batch D was obtained from a commercial company. The preparation procedure was not known.
